# A Mendelian randomization study on the causal association of circulating cytokines with colorectal cancer

**DOI:** 10.1371/journal.pone.0296017

**Published:** 2023-12-14

**Authors:** Youqian Kong, Xiaoyu Wang, Hongyun Xu, Shaoxuan Liu, Rui Qie

**Affiliations:** 1 Graduate School, Heilongjiang University of Chinese Medicine, Harbin, China; 2 Department of Internal Medicine, First Affiliated Hospital, Heilongjiang University of Chinese Medicine, Harbin, China; Kazi Nazrul University, INDIA

## Abstract

**Background:**

Circulating cytokines have been associated with colorectal cancer (CRC). However, their causal correlation remains undetermined. This investigation uses genetic data to evaluate the mechanism that links circulating cytokines and CRC *via* Mendelian Randomization (MR).

**Methods:**

A two-sample MR evaluation was carried out to investigate the mechanism associating circulating cytokines and CRC in individuals of European ancestry. The Genome-wide association studies statistics, which are publically accessible, were used. Eligible instrumental SNPs that were significantly related to the circulating cytokines were selected. Multiple MR analysis approaches were carried out, including Simple Mode, inverse variance weighted (IVW), MR-Egger, Weighted Mode, Weighted Median, and MR pleiotropy residual sum and outlier (MR-PRESSO) methods.

**Results:**

The evidence supporting the association of genetically predicted circulating levels with the increased risk of CRC was revealed; these included vascular endothelial growth factor (OR = 1.352, 95% CI: 1.019–1.315, *P* = 0.024), interleukin-12p70 (OR = 1.273, 95% CI: 1.133–1.430, *P* = 4.68×10^−5^), interleukin-13 (OR = 1.149, 95% CI: 1.012–1.299, *P* = 0.028), interleukin-10 (OR = 1.230, 95% CI: 1.013–1.493, *P* = 0.037), and interleukin-7 (OR = 1.191, 95% CI: 1.023–1.386 *P* = 0.024). Additionally, MR analysis negative causal association between macrophage colony stimulating factor and CRC (OR = 0.854, 95% CI: 0.764–0.955, *P* = 0.005). The data from Simple Mode, Weighted Median, MR-Egger, and Weighted Mode analyses were consistent with the IVW estimates. Furthermore, the sensitivity analysis indicated that the presence of no horizontal pleiotropy to bias the causal estimates.

**Conclusion:**

This investigation identified a causal association between circulating cytokines levels risk of CRC and may provide a deeper understanding of the pathogenesis of CRC, as well as offer promising leads for the development of novel therapeutic targets for CRC.

## Introduction

Colorectal cancer (CRC) is among the most frequently occurring diseases and a primary cause of increased mortality by cancer globally [1]. Currently, surgical resection is the primary CRC treatment strategy, combined with local pelvic radiation and systemic chemotherapy [2]. Immunotherapy is one of the new alternatives in cancer treatment, especially vaccines targeting cellular and humoral immune responses are expected to be a novel and effective strategy to intervene in CRC [3]. However, CRC therapies continue to confront significant obstacles. Approximately 1.8 million new cancer incidences were reported in 2018; one-third of these patients had metastasized stage [4]. Even though the industrialized countries have enhanced 5-year CRC patients’ survival rate due to early screening [5], according to the most recent statistics, the frequency of early CRC onset is increasing, particularly in young rectal cancer individuals [6]. Per previous research, in the United States, the CRC incidence rate is expected to be increased by 90% by 2030. Therefore, people are keen to find new treatment solutions in such a difficult condition [5]. Investigating the pathophysiology of CRC is beneficial for advancing the development of innovative treatment plans. Various immune cells and cytokines are involved in the incidence and development of CRC and are frequently linked with a chronic inflammatory state [7]. Inflammation is a physiological reaction to injury that begins with the release of biomolecules from injured tissues. The wound heals after white blood cell infiltration, but the signaling cascade continues in chronic inflammatory conditions [8]. Rudolf Virchow discovered the link between cancer and chronic inflammation more than 150 years ago [9]. Chronic inflammation causes epithelial-mesenchymal transition, dedifferentiation, increased amounts of reactive oxygen species and cytokines, and epigenetic alterations in tumor and stromal cells [10]. Furthermore, it contributes to carcinogenesis by causing gene mutations, blocking apoptosis, and increasing angiogenesis and cell proliferation [11]. Nuclear factor kappa B and cyclooxygenase-2 are critical inflammatory genes that establish a molecular connection between inflammation and cancer and are candidates for chemoprevention, particularly in CRC [12].

It has been indicated that CRC patients have altered cytokine levels. CRC patients have significant inflammatory infiltrates and enhanced cytokine expression in the tumor microenvironment. Toll-like receptors (TLRs) play a key role in this process. TLRs induce the production of pro-inflammatory mediators, activate inflammatory signaling cascades, and contribute to the formation of an inflammatory milieu. TLR2 and TLR4 affect immune homeostasis by regulating a variety of cytokines, such as IL-1, IL-6, IL-17A, and STAT3, which lead to inflammatory loss of control and CRC progression [13, 14]. Moreover, the levels of interferon by natural killer (NK) and T helper type 1 (Th1) CD4+, CD8+ cells limit tumor progression by activating cytotoxic immunity [15–17], and the presence of Th1 polarization markers correlate with lower tumor recurrence in CRC patients [18]. Conversely, transforming growth factor β (TGF-β) has been found to inhibit NK and CD8+ T-cell activity and reduce the expression of major histocompatibility complex (MHC) molecules on the surface of tumor cells, which helps cancer cells to evade immune surveillance and promotes the transformation of normal colon tissues to CRC [19, 20]. Tumor-specific upregulation of cytokines produced by Th17 CD4+ cells, such as IL-17A and IL-22, has been observed in human CRC [21–23], and studies in mouse models of spontaneous intestinal tumorigenesis have demonstrated the importance of these cytokines in tumor progression [24, 25].

Mendelian randomization (MR) is an analytic method that utilizes genetic variants to assess the causal association between a modifiable exposure or risk factor and a clinically relevant outcome [26]. Upon the satisfaction of instrumental variable criteria, the acquired estimator is consistent even in non-assessed confounding and reverse causation [27]. MR is more feasible than randomized controlled trials and reduces bias for confounding factors in observational studies. It has been applied to investigate the relationship between circulating cytokine and different diseases, such as Parkinson’s and Alzheimer’s, etc. [28, 29]. However, MR has not been used to explore the relationship between circulating cytokines and CRC. Hence, we employed a two-sample MR analysis to identify the possible causal link between circulating cytokines and CRC.

## Methodology

### Study design

**Fig 1:**
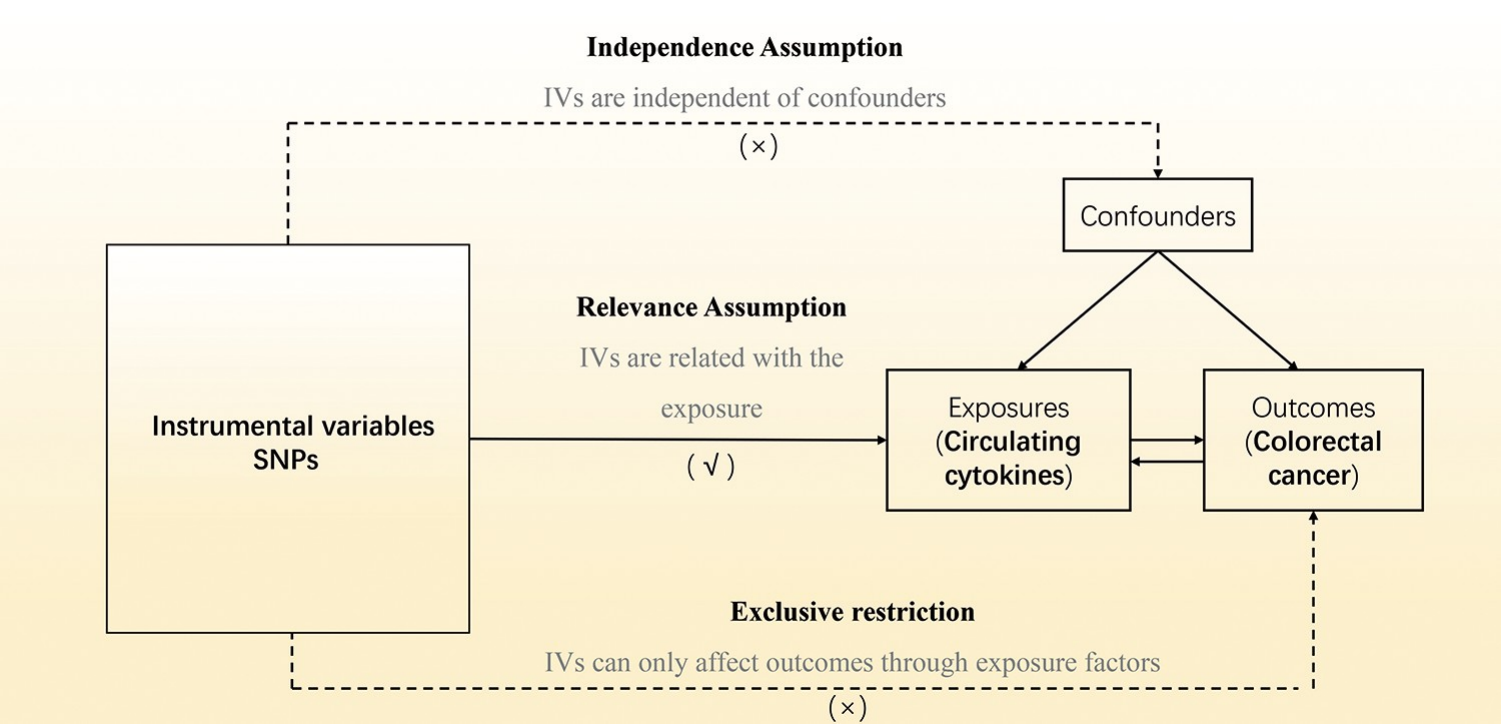
Overview of Mendelian randomization.

In our two-sample MR research (Fig 1), single nucleotide polymorphisms (SNPs) were utilized as IVs. To ensure the data validity, SNPs were chosen based on three major assumptions: (1) IVs should be substantially linked with the exposure factors (‘‘Relevance assumption”); (2) IVs should affect the outcomes only *via* exposure factors and not by other pathways, which implies no horizontal pleiotropy (‘‘Exclusivity assumption”); (3) IVs should not be relevant to any confounding factors (‘‘Independence assumption”) [30].

### Data resource

The SNPs on circulating cytokines and CRC were selected from the latest GWAS, as depicted in S1 Table.

Fig 1 depicts the study’s overview in detail. Summary data from the most thorough genome-wide association study (GWAS) cytokine was used for cytokines’ genetic tool. The GWAS cytokine meta-analysis comprised 8,293 Finns from 3 distinct population-dependent cohorts: FINRISK1997, Young Finns Cardiovascular Risk, and FINRISK2002 studies [31]. Participants were randomly chosen from five distinct geographic regions and between the ages of 25 and 74 during the survey’s administration in cross-sectional surveys conducted in 1997, 2002, and 2015. The subjects’ blood, EDTA plasma, and heparin plasma were tested for cytokine levels. Only observations within each cytokine’s detectable range were included, and cytokines with > 90% missing values were removed from the study. (7 out of 48). Each subject gave written informed permission.

To explore the causal effect of various circulating cytokines on the risk of CRC, we selected datasets for CRC as the outcome from the summary statistics of the GWAS from FinnGen consortium [32] (https://www.finngen.fi/en) data version 5 (R5 release version 11 May 2021), this GWAS included 3,022 CRC cases and 215,770 controls. Then, using GWAS summary statistics, we used two-sample MR methods to deduce the causative link between inflammatory factors and CRC. Since samples of inflammatory regulators and CRC were acquired from various consortiums, there was no overlap.

### SNPs selection

We performed a set of methods to filter valid SNPs that suit the three core MR assumptions. Firstly, the independent SNPs strongly linked to different circulating cytokines were selected [33, 34]. To obtain more SNPs as IVs, those with *P*-value < 5×10^−6^ were considered to be significantly associated with circulating cytokines. Secondly, we adopted the clumping process to evaluate the linkage disequilibrium (LD) among the SNPs (r^2^ < 0.001 and clumping distance = 10,000kb). The SNPs with LD were removed to avoid biased results. Thirdly, all the screened SNPs were searched on PhenoScanner V2 (http://www.phenoscanner.medschl.cam.ac.uk/) [35]. PhenoScanner V2 provides the phenotypes information of SNPs, which can be used to determine whether the SNPs only affect the outcomes through exposure. The SNPs related to confounding factors, such as smoking, diabetes, and worries, were excluded to eliminate the bias. Lastly, we harmonized the exposure and outcome datasets to remove the non-concordant SNPs. The remaining SNPs were used as the genetic IVs.

Moreover, the F statistics for the SNPs were assessed as follows: *F* = *R*^2^×(*N*−2)/(1−*R*^2^), R^2^ = proportion of variance. N = sample size. Weak instruments were identified by IVs with an F statistic less than 10 (F < 10) and excluded from the analysis [36].

### Statistical analysis

After selecting the valid SNPs, we adopted inverse variance weighted (IVW) as the main way to estimate the MR analysis. IVW assesses the overall causal impact of exposure on the outcomes. It is the most accurate way to evaluate causality if all the selected SNPs are valid [37]. We also applied complementary methods to analyze causal association, including Weighted Median, MR Egger, Weighted Mode, and Simple Mode methods. The Weighted Median method will generate a more potent effect when more than half of the SNPs are valid [38]. MR Egger provides accurate effect estimates even if all the SNPs are invalid [36].

We further conducted the MR-Egger regression and the MR Pleiotropy Residual Sum and Outlier (MR-PRESSO) test to evaluate the possible horizontal pleiotropy [39, 40]. In the MR-Egger regression, the intercept term indicates the average pleiotropic effect of IVs [40]. We used Cochran’s Q statistic and MR-egger regression to test the heterogeneities. Additionally, the leave-one-out analysis was utilized to assess the robustness and consistency of the results.

All the analyses were performed with the packages “Two Sample MR” and “MRPRESSO” in R version 4.2.1. and the statistical significance threshold was established to be *P* < 0.05.

## Results

### Causal effect of circulating cytokines on CRC

After the series of filters mentioned in the method, 4–16 SNPs were left as IVs for circulating cytokines (S1 Table). All the selected SNPs were robust instruments, as confirmed by the F-statistic values being more than 10.

We adopted these SNPs to analyze the causal link. The MR estimates between circulating cytokines and CRC of different methods are presented in S2 Table. The preliminary results of IVW revealed positive causal effect of 6 cytokines on CRC (Table 1), including vascular endothelial growth factor (VEGF) (OR = 1.352, 95% CI: 1.019–1.315, P = 0.024), interleukin-12p70 (IL-12p70) (OR = 1.273, 95% CI: 1.133–1.430, P = 4.68×10–5), interleukin-13 (IL-13) (OR = 1.149, 95% CI: 1.012–1.299, P = 0.028), interleukin-10 (IL-10) (OR = 1.230, 95% CI: 1.013–1.493, P = 0.037), and interleukin-7 (IL-7) (OR = 1.191, 95% CI: 1.023–1.386 P = 0.024), and negative causal effect of macrophage colony stimulating factor (M-CSF) (OR = 0.854, 95% CI: 0.764–0.955, P = 0.005). Furthermore, the MR-Egger and Weighted Median indicated consistent results. Noteworthy, the relationship between IL-13 and CRC should be carefully investigated as the Simple mode method yielded an inverse association compared to the other MR methods. The scatter plots demonstrated the specific effects of each method per outcome database (S1 Fig).

**Table 1:** MR estimates for the association between circulating cytokines and CRC with the IVW method.

Cytok (exposure)	MR method	No.SNP	*Beta*	SE	P-value	OR	95%CI
Vascular endothelial growth factor levels	MR Egger	12	0.299	0.112	0.028	1.349	1.084–1.679
	Weighted median	12	0.210	0.053	0.000	1.233	1.112–1.368
	Inverse variance weighted	12	0.160	0.072	0.026	1.173	1.019–1.351
	Simple mode	12	0.234	0.142	0.134	1.263	0.957–1.668
	Weighted mode	12	0.213	0.057	0.005	1.238	1.107–1.384
Macrophage colony stimulating factor levels	MR Egger	9	-0.052	0.100	0.621	0.949	0.781–1.154
	Weighted median	9	-0.084	0.077	0.273	0.920	0.791–1.068
	Inverse variance weighted	9	-0.158	0.057	0.005	0.854	0.764–0.955
	Simple mode	9	-0.063	0.121	0.620	0.939	0.742–1.190
	Weighted mode	9	-0.056	0.103	0.603	0.946	0.773–1.156
Interleukin-12p70 levels	MR Egger	14	0.333	0.100	0.010	1.396	1.148–1.697
	Weighted median	14	0.267	0.065	0.000	1.306	1.150–1.484
	Inverse variance weighted	14	0.242	0.059	0.000	1.273	1.133–1.430
	Simple mode	14	0.155	0.146	0.318	1.167	0.876–1.555
	Weighted mode	14	0.264	0.074	0.006	1.302	1.126–1.504
Interleukin-13 levels	MR Egger	12	0.249	0.119	0.074	1.283	1.016–1.620
	Weighted median	12	0.207	0.063	0.001	1.230	1.087–1.393
	Inverse variance weighted	12	0.139	0.063	0.028	1.149	1.015–1.299
	Simple mode	12	-0.151	0.185	0.439	0.860	0.599–1.236
	Weighted mode	12	0.247	0.064	0.005	1.280	1.129–1.450
Interleukin-10 levels	MR Egger	11	0.574	0.161	0.007	1.775	1.295–2.434
	Weighted median	11	0.293	0.090	0.001	1.340	1.123–1.598
	Inverse variance weighted	11	0.207	0.099	0.037	1.230	1.013–1.493
	Simple mode	11	0.246	0.237	0.327	1.279	0.803–2.037
	Weighted mode	11	0.346	0.091	0.004	1.414	1.183–1.690
Interleukin-7 levels	MR Egger	12	0.464	0.171	0.030	1.591	1.137–2.225
	Weighted median	12	0.250	0.075	0.001	1.284	1.109–1.487
	Inverse variance weighted	12	0.174	0.077	0.024	1.191	1.023–1.386
	Simple mode	12	0.225	0.133	0.131	1.252	0.964–1.626
	Weighted mode	12	0.285	0.082	0.008	1.330	1.133–1.560

**Fig 2:**
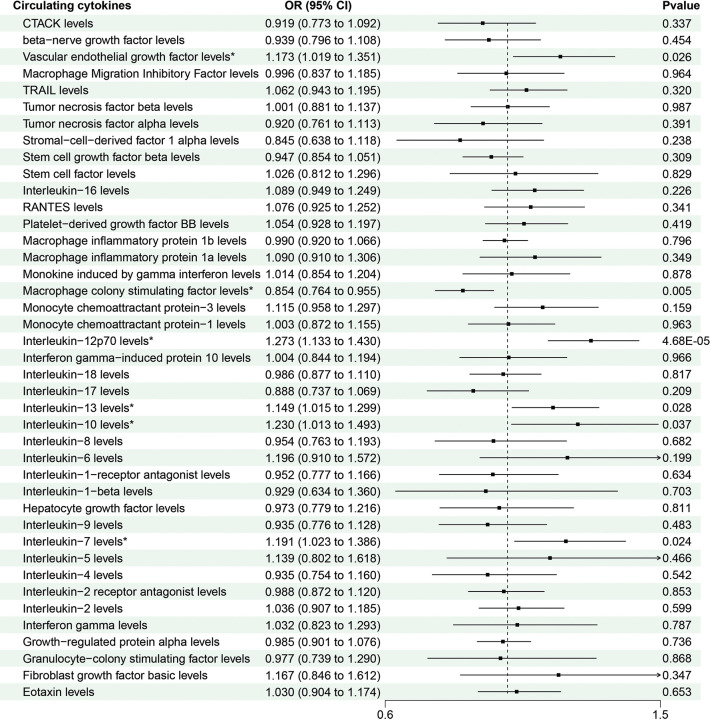
MR results of circulating cytokines on risk of colorectal cancer based on the IVW model.

Apart from VEGF, IL-12p70, IL-13, IL-10, IL-7and M-CSF, the other 35 cytokines (e.g., GRO-α, Trail, MIG, IL -17) did not show any association with the risk of CRC in either IVW primary MR analysis or in other secondary analyses (Fig 2). In the heterogenity and pleiotropy analyses, Cochran’s Q statistic and MR-egger regression showed no heterogeneity between the individual SNPs (P > 0.05) (Table 2). An additional solidity test, The p-values of the MR PRESSO global test for circulating cytokines on CRC were all greater than 0.05 (S3 Table). Additionally, the leave-one-out analysis further confirmed the causal estimates of circulating cytokines (S2 Fig).

**Table 2:** Heterogenity and pleiotropy analyses for associations between circulating cytokines and CRC.

cytokines (exposure)	Heterogenity		MR-Egger intercept		
	Q	Q_pval	egger_intercept	se	pval
CTACK levels	8.772	0.187	-0.065	0.037	0.127
beta-nerve growth factor levels	3.758	0.585	0.081	0.061	0.240
Vascular endothelial growth factor levels	16.095	0.061	-0.042	0.027	0.157
Macrophage Migration Inhibitory Factor levels	1.275	0.866	-0.057	0.040	0.226
TRAIL levels	17.473	0.095	-0.020	0.022	0.386
Tumor necrosis factor beta levels	0.238	0.888	0.015	0.030	0.675
Tumor necrosis factor alpha levels	0.840	0.840	-0.060	0.033	0.171
Stromal-cell-derived factor 1 alpha levels	3.302	0.653	0.048	0.028	0.147
Stem cell growth factor beta levels	12.281	0.423	-0.031	0.021	0.165
Stem cell factor levels	9.730	0.204	0.026	0.035	0.482
Interleukin-16 levels	14.653	0.066	0.015	0.033	0.667
RANTES levels	4.455	0.726	-0.084	0.041	0.080
Platelet-derived growth factor BB levels	6.279	0.854	0.011	0.019	0.574
Macrophage inflammatory protein 1b levels	10.518	0.786	0.020	0.017	0.250
Macrophage inflammatory protein 1a levels	0.266	0.992	0.015	0.044	0.744
Monokine induced by gamma interferon levels	29.214	0.004	-0.019	0.046	0.686
Macrophage colony stimulating factor levels	4.312	0.634	-0.044	0.034	0.244
Monocyte chemoattractant protein-3 levels	0.206	0.650	0.027	0.073	0.774
Monocyte chemoattractant protein-1 levels	9.761	0.552	-0.030	0.023	0.221
Interleukin-12p70 levels	4.123	0.846	-0.021	0.018	0.286
Interferon gamma-induced protein 10 levels	7.095	0.419	-0.054	0.026	0.078
Interleukin-18 levels	20.268	0.089	-0.038	0.028	0.203
Interleukin-17 levels	2.174	0.950	-0.014	0.030	0.643
Interleukin-13 levels	10.845	0.146	-0.034	0.031	0.311
Interleukin-10 levels	9.606	0.294	-0.056	0.022	0.032
Interleukin-8 levels	2.224	0.329	-0.013	0.043	0.788
Interleukin-6 levels	0.764	0.858	-0.066	0.040	0.195
Interleukin-1-receptor antagonist levels	0.871	0.832	-0.037	0.044	0.461
Interleukin-1-beta levels	1.976	0.372	0.045	0.044	0.415
Hepatocyte growth factor levels	2.593	0.762	0.067	0.040	0.153
Interleukin-9 levels	2.013	0.733	0.054	0.051	0.355
Interleukin-7 levels	10.411	0.166	-0.086	0.047	0.108
Interleukin-5 levels	9.736	0.021	0.091	0.072	0.300
Interleukin-4 levels	4.427	0.730	0.009	0.033	0.801
Interleukin-2 receptor antagonist levels	0.478	0.976	-0.055	0.030	0.143
Interleukin-2 levels	5.139	0.643	0.001	0.023	0.958
Interferon gamma levels	1.198	0.977	0.005	0.029	0.860
Growth-regulated protein alpha levels	2.992	0.810	0.007	0.033	0.835
Granulocyte-colony stimulating factor levels	11.305	0.079	0.024	0.034	0.502
Fibroblast growth factor basic levels	0.138	0.933	0.069	0.081	0.484
Eotaxin levels	7.801	0.856	0.019	0.022	0.408

### No causal effect of CRC on circulating cytokines

**Fig 3:**
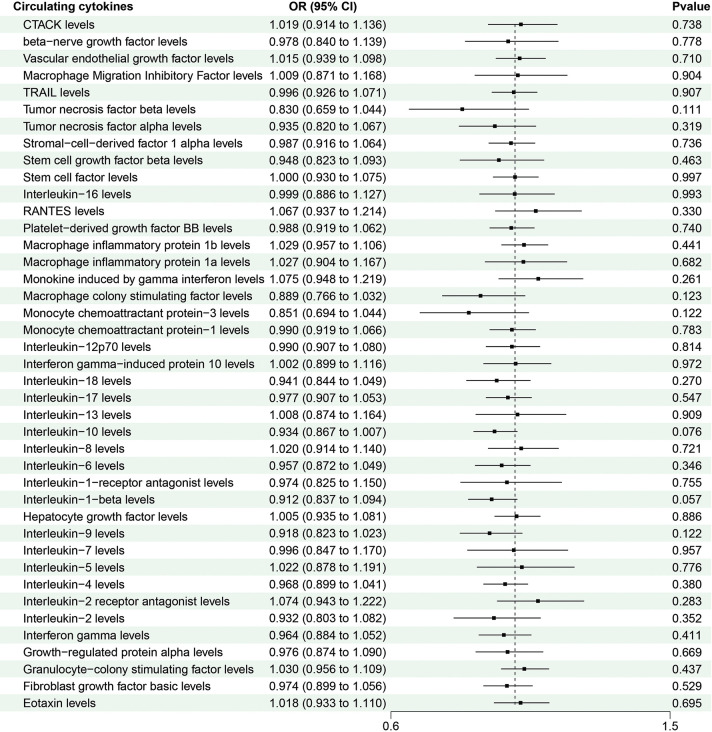
MR results of colorectal cancer on circulating cytokines based on the IVW model.

To further explore the causal effect of CRC on the significant circulating cytokines, we selected independent SNPs as IVs for CRC. The F-statistic values were all higher than 10, which confirmed that all the selected SNPs were valid instruments. The results of the IVW analysis confirmed no causal effect of CRC on all circulating cytokines (Fig 3). Furthermore, it revealed no heterogeneities based on the results of Cochran’s Q statistic. Horizontal pleiotropy was not detected in the results of several cytokines based on the results of MR-Egger intercept (P > 0.05) and MR-PRESSO global test (P > 0.05). The detailed data are shown in S4 and S5 Tables.

## Discussion

This is the first MR study to explore the causal association between circulating cytokines and CRC. The MR analysis showed no bidire ctional genetic liability between cytokines and CRC. These results should be cautiously interpreted as MR is a specific study focusing on data resources based on genetic variants.

In this bidirectional MR study, we discovered that higher levels of genetically determined M-CSF may reduce risk of CRC while higher levels of genetically determined VEGF, IL-12p70, IL-13, IL-10, and IL-7 were linked with increased risk of CRC. Moreover, our findings showed that no altered levels of circulating cytokines were directly linked to CRC.

The GM-CSF and M-CSF have been associated with the polarization of pro-inflammatory/antitumor M1 and anti-inflammatory/pro-angiogenic M2 macrophages [41, 42]. It was demonstrated that after *in-vitro* GM-CSF stimulation but not with M-CSF, GM-CSF down-regulates major recognition receptors on human monocytes, including TLR1, TLR2, and TLR4, impeding proinflammatory cytokine production in the TLR axis, resulting in the peripheral blood monocytes exert cytostatic impact on CRC cells [13]. Furthermore, the assessment of >40 matched malignant CRC tissues indicated enhanced expression of the GM-CSF gene than the autologous healthy mucosa. Therefore, CRC tissues have a cytokines’ gene expression pattern similar to, but not identical to, activated M1 cells, including increased gene expression of IL1 and IL23 [41]. The increased inflammatory responses noticed during wound healing may partly be caused by the high expression of M-CSF in CRC. This MR investigation also revealed that increased circulating M-CSF levels were linked with a reduced risk of CRC (95% CI = 0.764–0.955, OR = 0.854, *P* = 0.005, per 1 Standard deviation increase).

Furthermore, our MR analysis revealed a potential association between several interleukins and the increased risk of CRC. IL-10 is an important immune suppressor, and reduced IL-10R in colorectal tissue might cause severe spontaneous colitis, increasing the risk of CRC initiation [43]. However, the significance of IL-10 in cancer etiology and development is complicated. Lentivectors expressing shRNA specific to IL-10 (shIL-10 LVs) repressed IL-10 expression and decreased CRC development when combined with CY. Furthermore, IL-10 absence enhanced the efficiency of DC-based immunotherapy decreased Treg and MDSC levels in the tumor microenvironment, and boosted Th1-type antitumor responses, indicating that IL-10 promotes tumor growth in CRC [44]. Another investigation revealed that when a mouse tumor model was treated with genetically modified lactic acid bacteria designed to generate IL-10 or antioxidant enzymes, the CRC tumor development was inhibited [45], demonstrating that IL-10 might limit tumor progression. IL-10 expression was shown to be lower in patients 7 days after CRC surgery than before, and patients with recurrence CRC after surgery had substantially greater levels of IL-10, showing that IL-10 can be used as a predictive biomarker in CRC [46]. Nonetheless, whether IL-10 promotes or inhibits tumor growth has to be determined.

Non-hematopoietic stromal and dendritic cells generate IL-7, where later only produce a small amount [47]. Although CRC patients indicated increased IL-7 levels than the controls, and the expression was associated with metastatic disease and tumor location [48, 49], little is known about the fundamental causes of IL-7-induced CRC aggression. Therefore, further progress should be made to investigate its biological role in CRC and associated molecular interaction between IL-7 and other immune system components. In our study, several cytokines suggested the association with CRC. However, there was little previous evidence for IL-12p70 and IL-13, and more studies on them are warranted.

Apart from interleukins, previous studies sought to identify the profiles of various chemokines implicated in CRC patients to assess their involvement in the etiology of the disease. Tumor cell immune escape requires several phases, linkages, and variables. Immune cells, including MDSC-mediated negative immune regulation and faulty antitumor T cells, are directly implicated in the body’s antitumor immunological response and tumor immune escape [50–52]. Since there are many links between immune system and chemokines, chemokines play a significant role in immune evasion. Increased concentrations of activated CD8+ T lymphocytes infiltrate tumor sites as a protector in organisms and is associated with a better prognosis in CRC patients. However, in patients with advanced malignancy, activated CD8+ T cells are considerably diminished [53]. CXCR3 is expressed on the surface of CD8+ T lymphocytes, and its ligands are CXCL9, 10, and 11. Therefore, in response to those chemokines, CD8+ T lymphocytes are attracted to the tumor site and play an effective anti-cancer function [54, 55]. Neutrophils are distinct from the lineage of myelocytic. CXCR2 interacts with its ligands, including CXCL1, 2, 5, 7, and 8, and is responsible for neutrophil recruitment [56–58]. Because of the formation of a differentiated phenotype, neutrophils, like macrophages, play distinct functions in tumor immune response. It has been indicated that neutrophils are tumor-cytotoxic; however, some investigations contradict this and reveal that neutrophils are involved in tumor cell metastasis. Furthermore, neutrophil counts and neutrophil-related variables are linked to cancer progression [59, 60].

Our research has several advantages. (1) This is the first MR research to explain how inflammatory mediators and the likelihood of CRC interact. (2) Unlike epidemiologic studies, our current investigation minimizes the possibility of confounding variables and reverse causation, yielding a valid set of cause-and-effect relationships. (3) The huge quantity of original research data in the GWAS database, which is open to the public, served as a reliable assurance for this study. (4) The time and money spent on this research were very cost-effective for the outcomes we found, in contrast to the time-consuming RCTs.

Interpreting our results requires taking into account their limitations. We did not address CRC complications in our research, which would have been preferable given the etiologic and prognostic heterogeneity within each clinical symptom, aside from the intrinsic flaws related to the validity of required assumptions underpinning causal interpretation within MR studies. Such analyses would be constrained by the tiny sample size and weak statistical strength. In any real clinical scenario, unexpected variables may also have an impact on variations in inflammatory factors. Additionally, MR could evaluate the long-term impacts of genetically anticipated inflammatory factors, but adult life is inhibited directly due to numerous unidentified factors. We removed the SNPs linked with mortality that were proxied by age at enrollment in order to reduce these biases. Finally, cytokines are a dynamic indicator, and MR does not take into account the changes in cytokine levels, in contrast to other indicators like weight.

## Conclusion

Our study elucidated a causal association between circulating levels of M-CSF, VEGF, IL-12p70, IL-13, IL-10, and IL-7 and risk of CRC, and may provide a deeper understanding of the pathogenesis of CRC, as well as the development of effective management strategies for the clinic. We suggest that M-CSF, VEGF, IL-12p70, IL-13, IL-10, and IL-7 may serve as potential therapeutic targets for CRC development.

## Supporting information

S1 Table Characteristics of the genetic instrument variables for cytokines. (XLSX)

S2 Table MR analysis of risk of colorectal cancer. (XLSX)

S3 Table Global test of MRPRESSO analysis. (XLSX)

S4 Table Heterogenity and pleiotropy analyses (reverse). (XLSX)

S5 Table Global test of MRPRESSO analysis (reverse). (XLSX)

S1 Fig Leave-one-out analysis further confirmed the causal estimates of circulating cytokines. (DOCX)

S2 Fig Scatter plots demonstrated the specific effects of each method per outcome database. (DOCX)
