# Supplementary material for: A Mendelian randomization study on the causal association of circulating cytokines with colorectal cancer
Source: PLoS One. 2023 Dec 14;18(12):e0296017. doi: 10.1371/journal.pone.0296017 (PMC10721084; doi:10.1371/journal.pone.0296017)
Supplement: S1 Fig — (DOCX) [file pone.0296017.s006.docx]

**Supplementary Figure S1.** Scatter plots demonstrated the specific effects of each method per outcome database


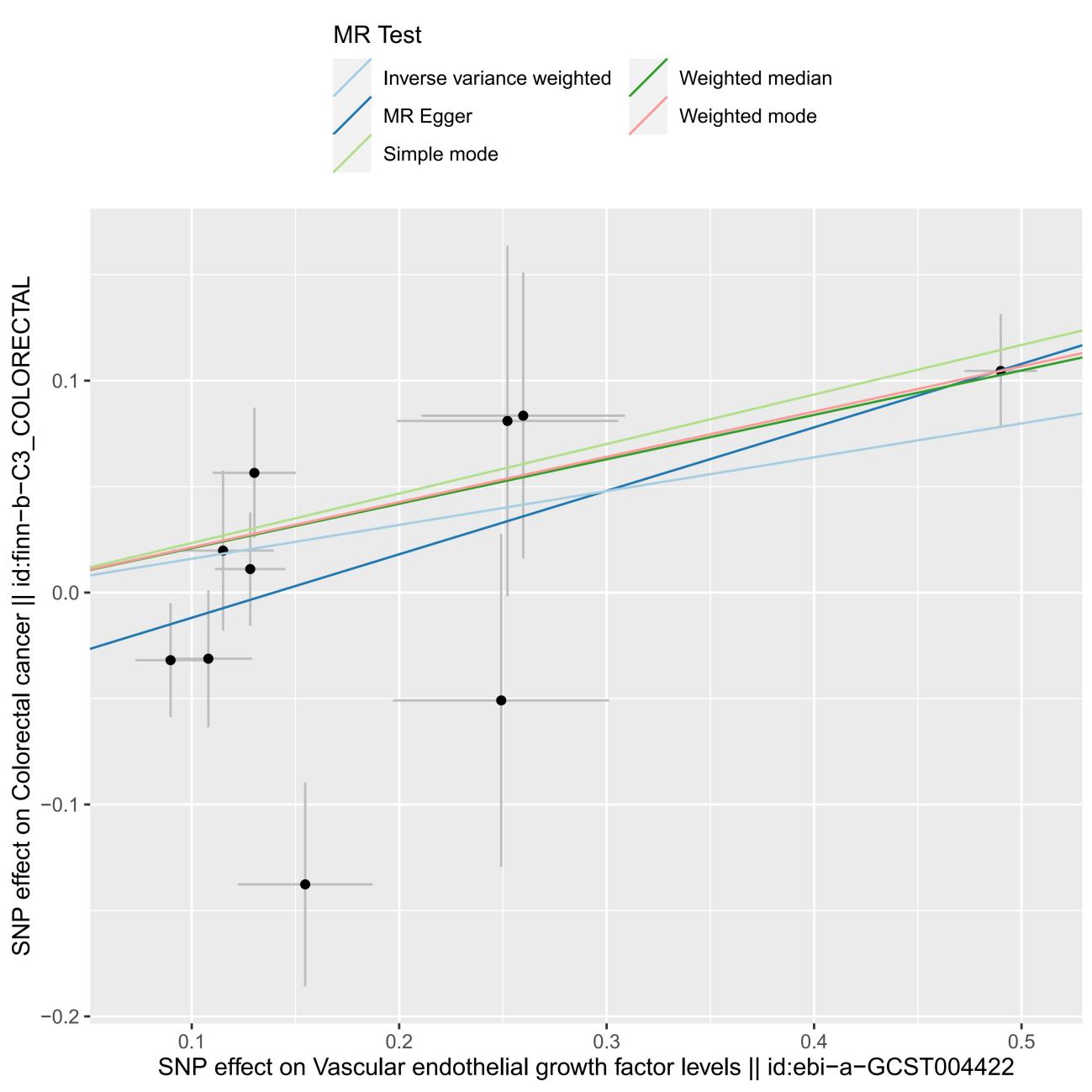


A. scatter plot of Vascular endothelial growth factor levels


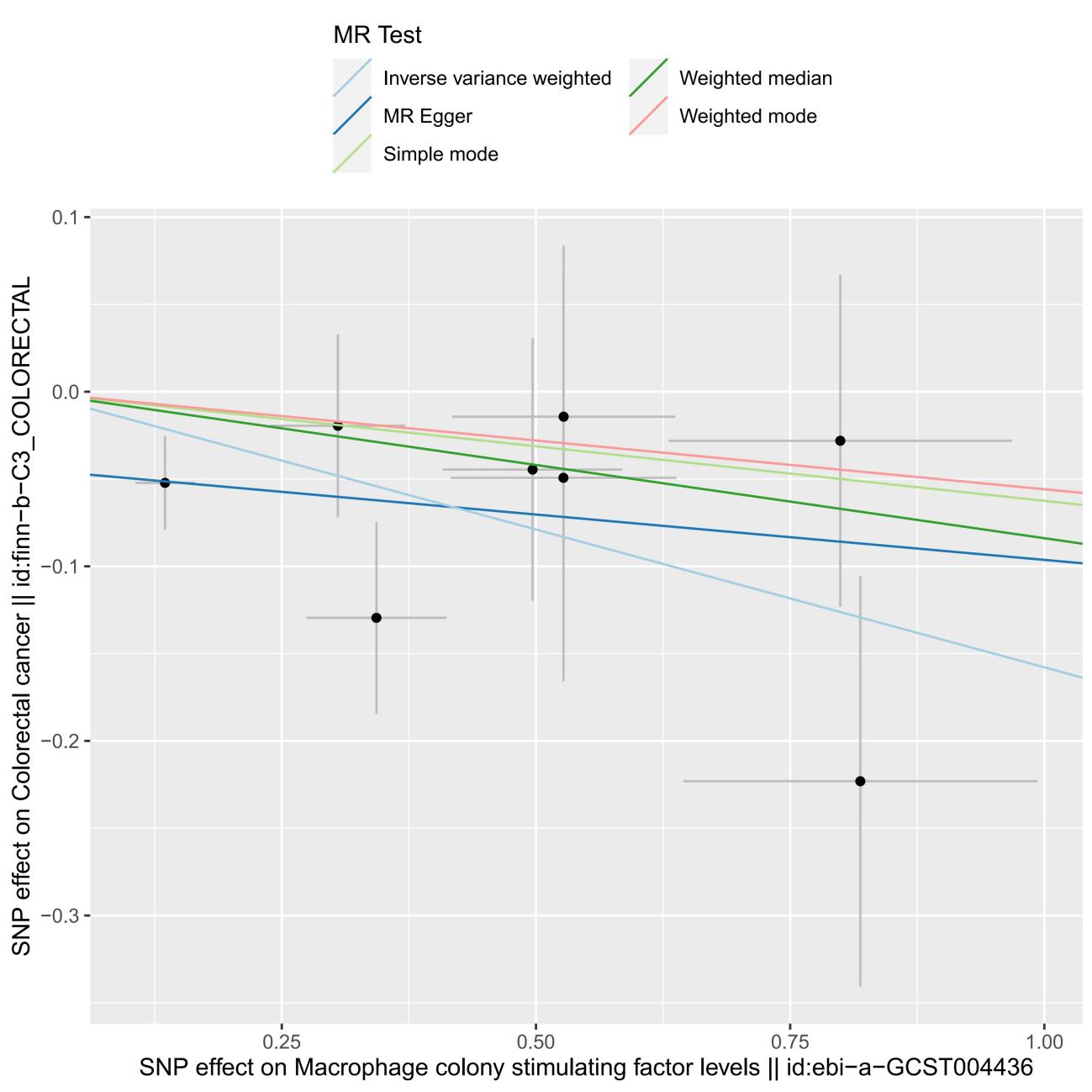


B. scatter plot of Macrophage colony stimulating factor levels


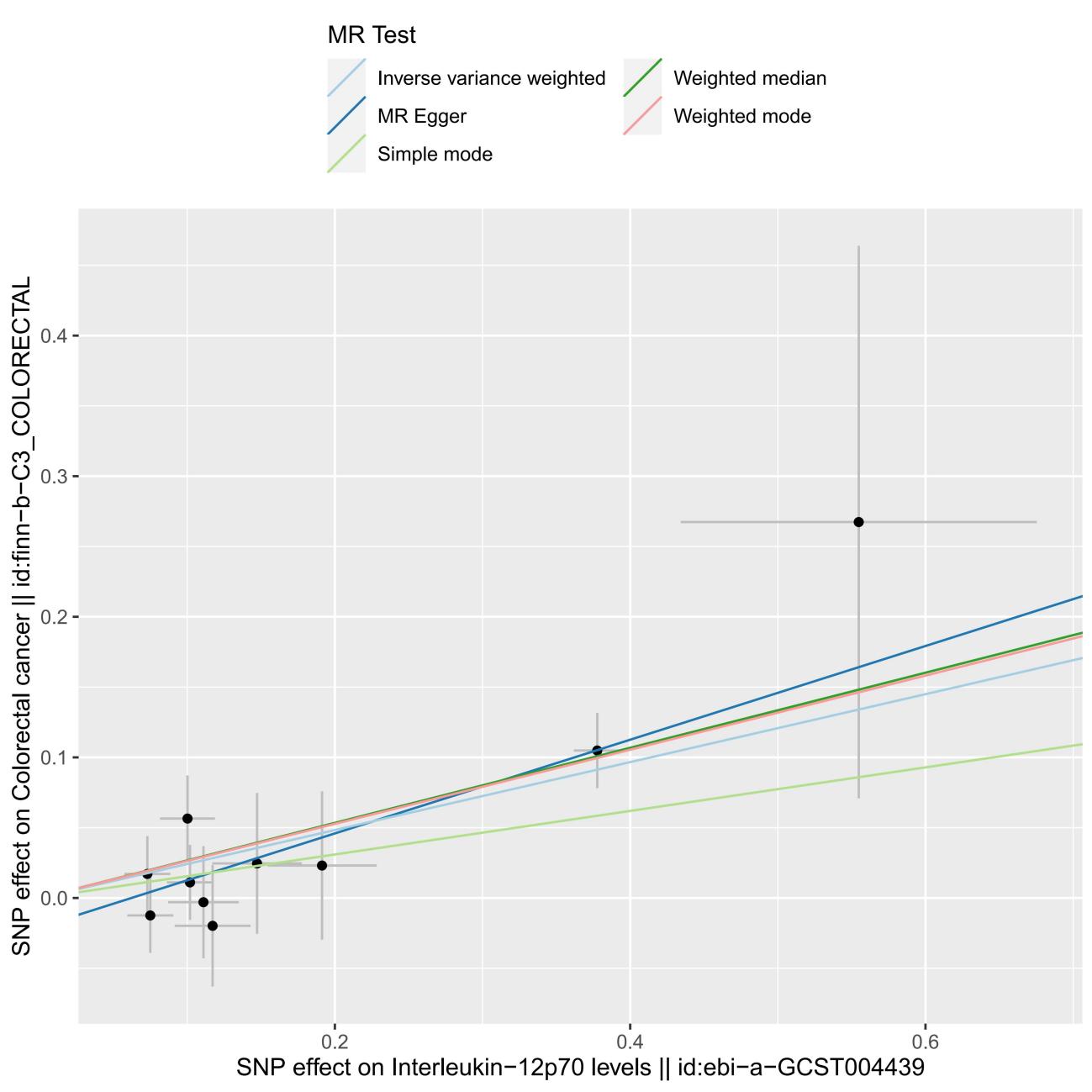


C. scatter plot of Interleukin-12p70 levels


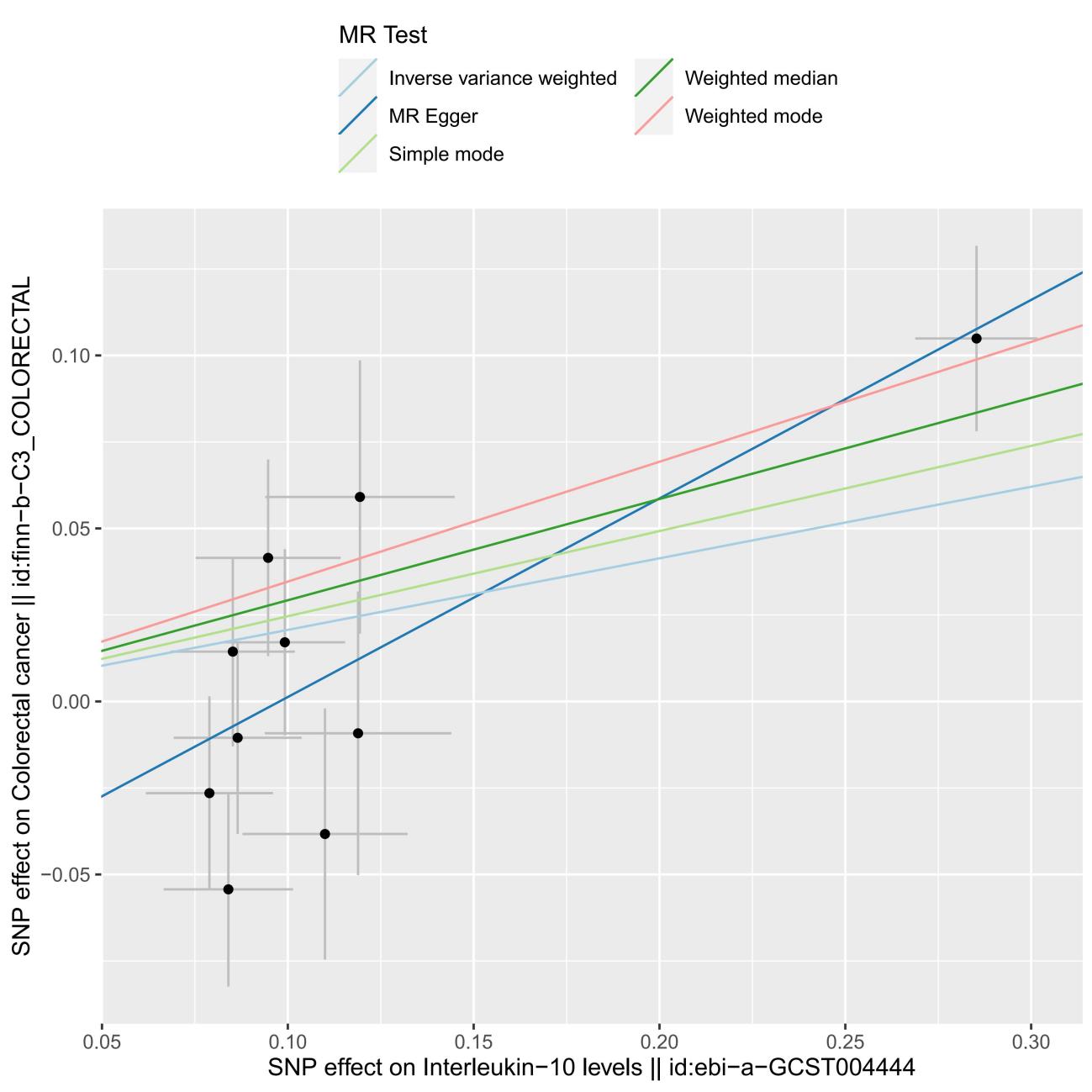


D. scatter plot of Interleukin-10 levels


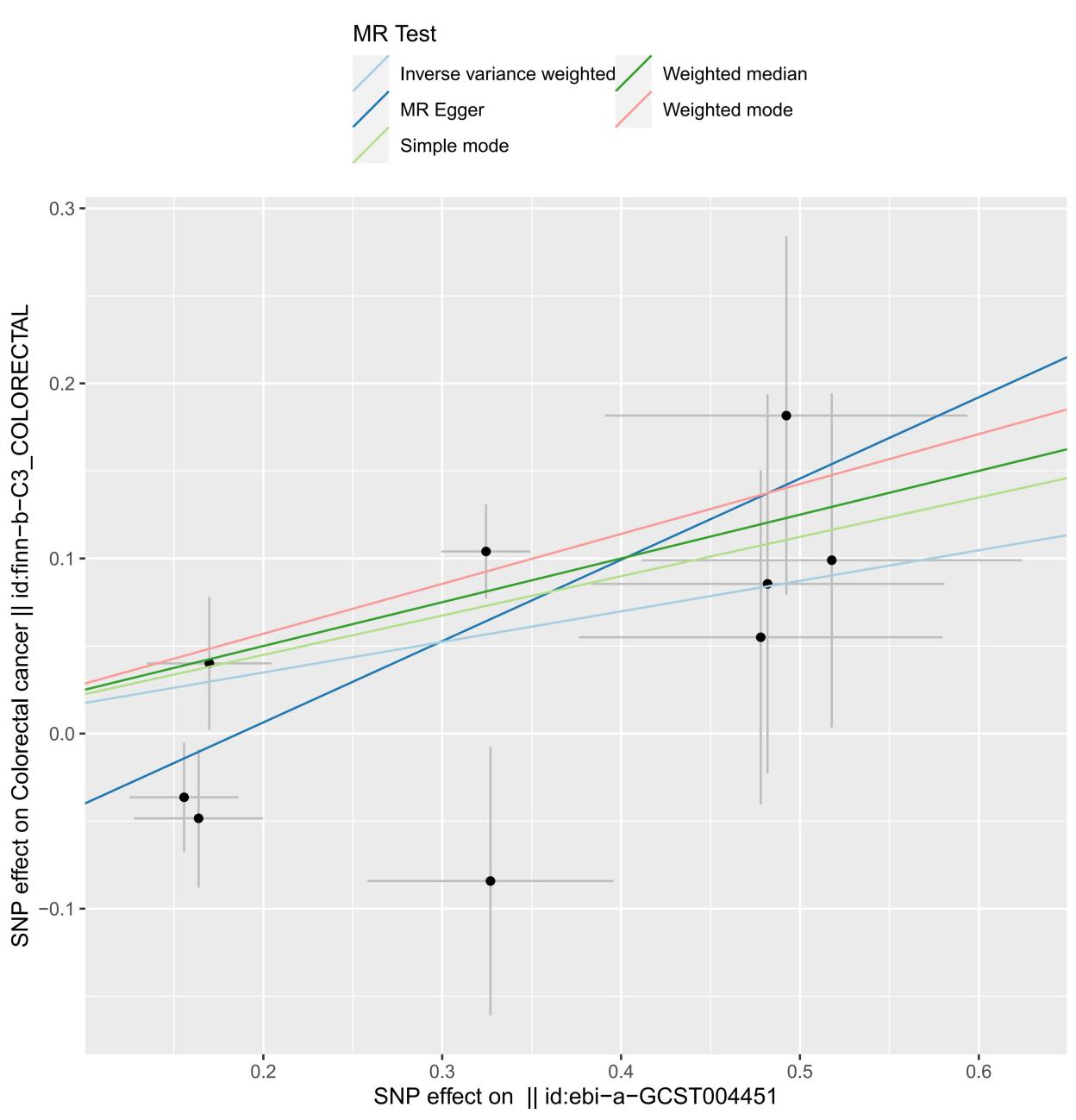


E. scatter plot of Interleukin-7 levels


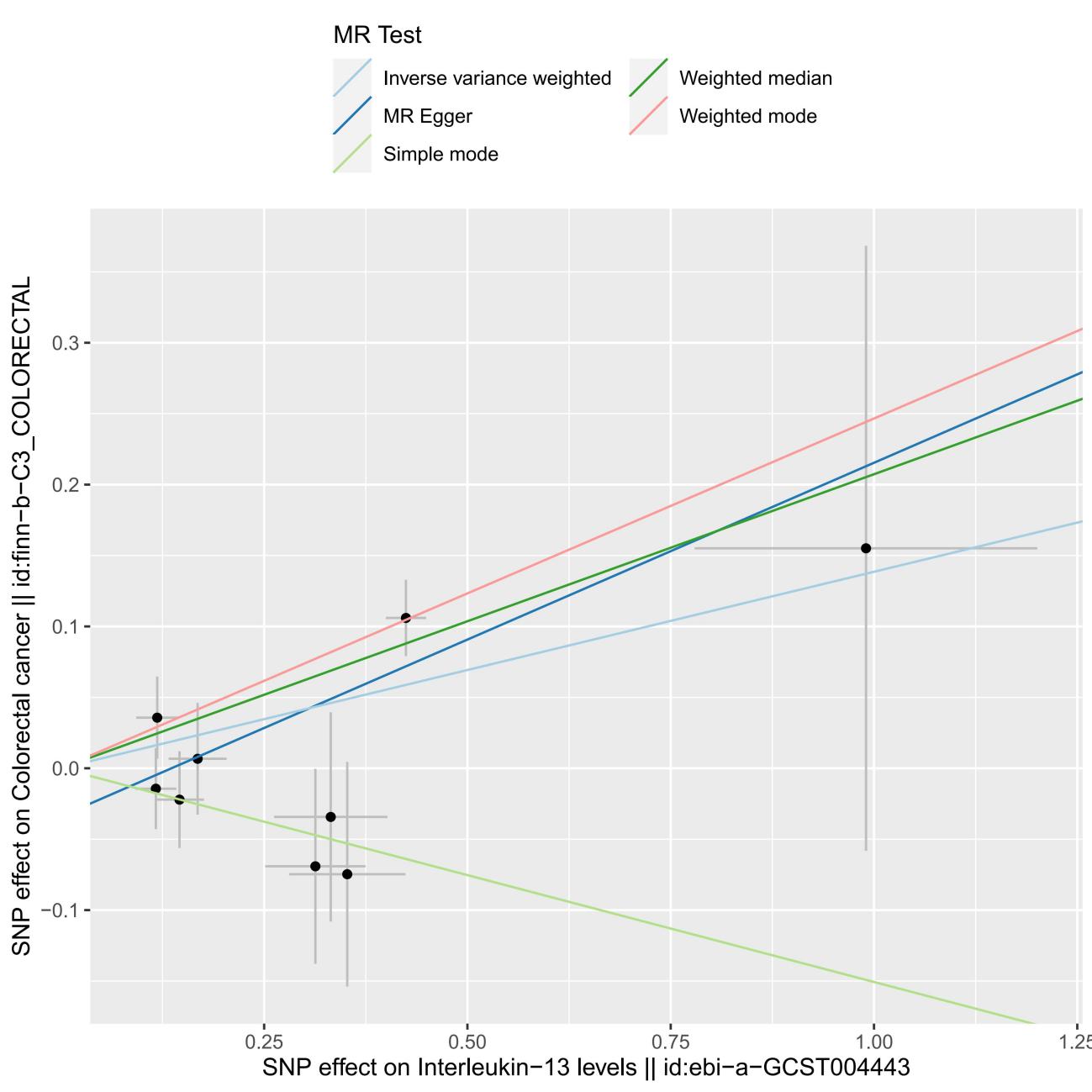


F. scatter plot of Interleukin-13 levels
