# Supplementary material for: A Mendelian randomization study on the causal association of circulating cytokines with colorectal cancer
Source: PLoS One. 2023 Dec 14;18(12):e0296017. doi: 10.1371/journal.pone.0296017 (PMC10721084; doi:10.1371/journal.pone.0296017)
Supplement: S2 Fig — (DOCX) [file pone.0296017.s007.docx]

**Supplementary Figure S2.** Leave-one-out analysis further confirmed the causal estimates of circulating cytokines


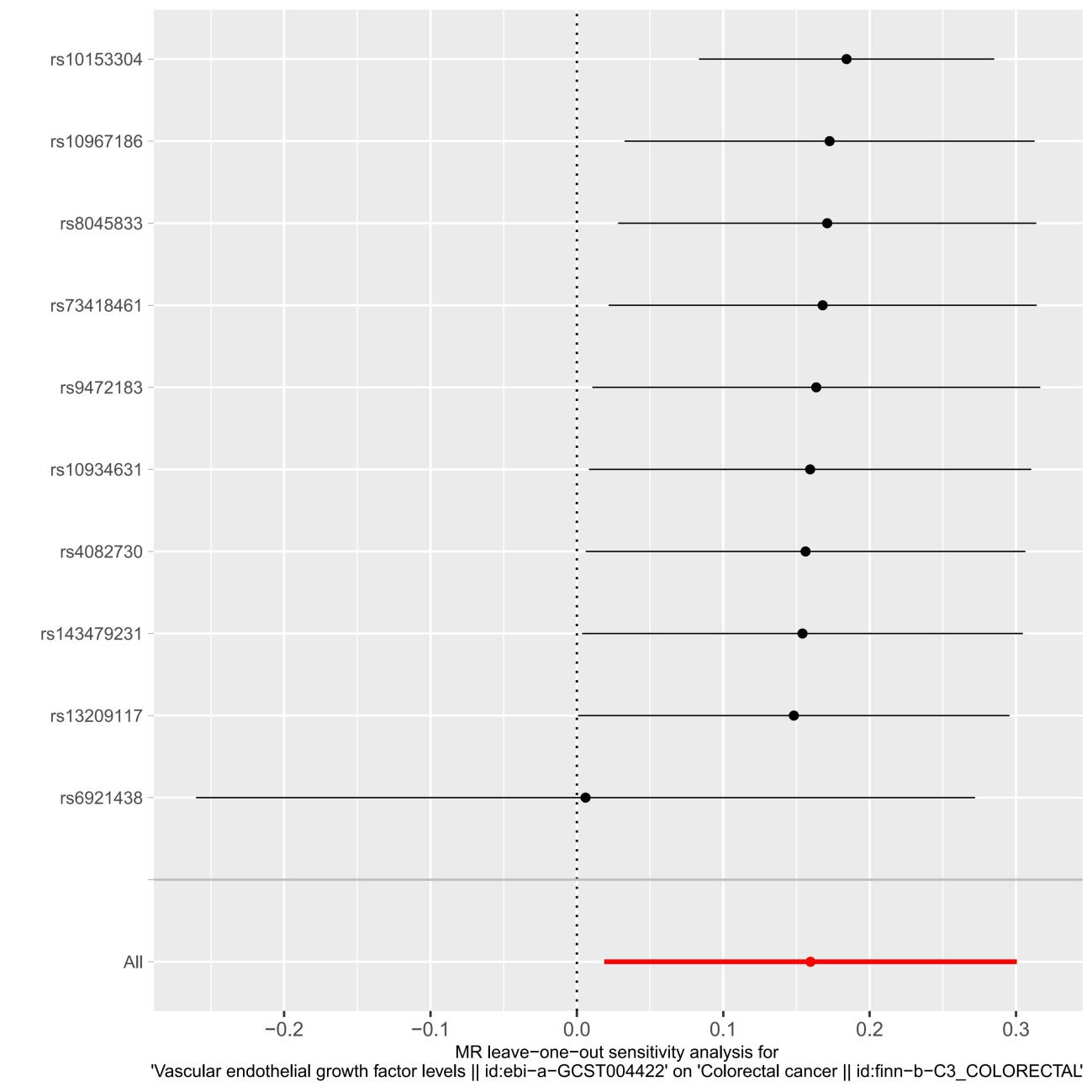


A. Leave-one-out plot of Vascular endothelial growth factor levels


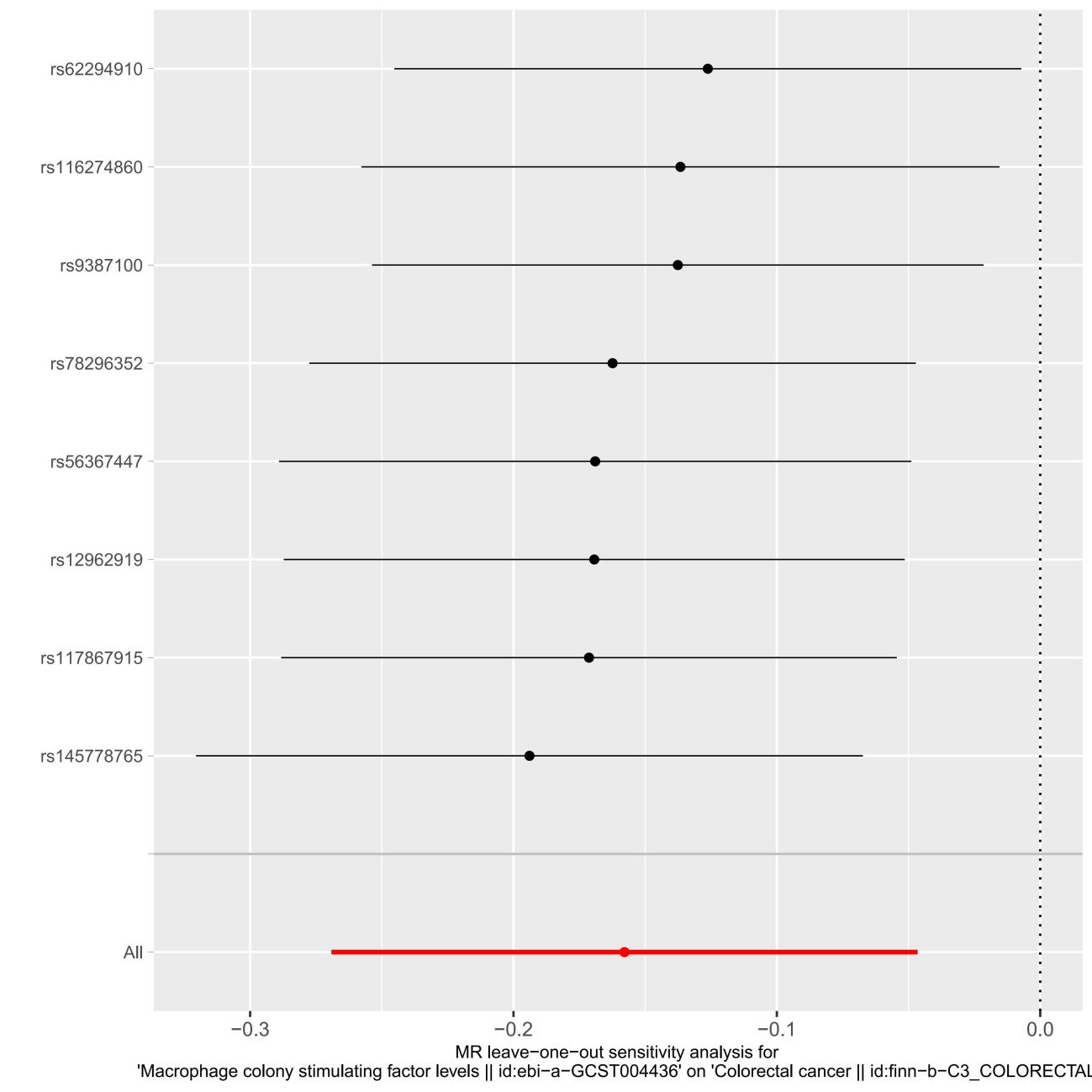


B. Leave-one-out plot of Macrophage colony stimulating factor levels


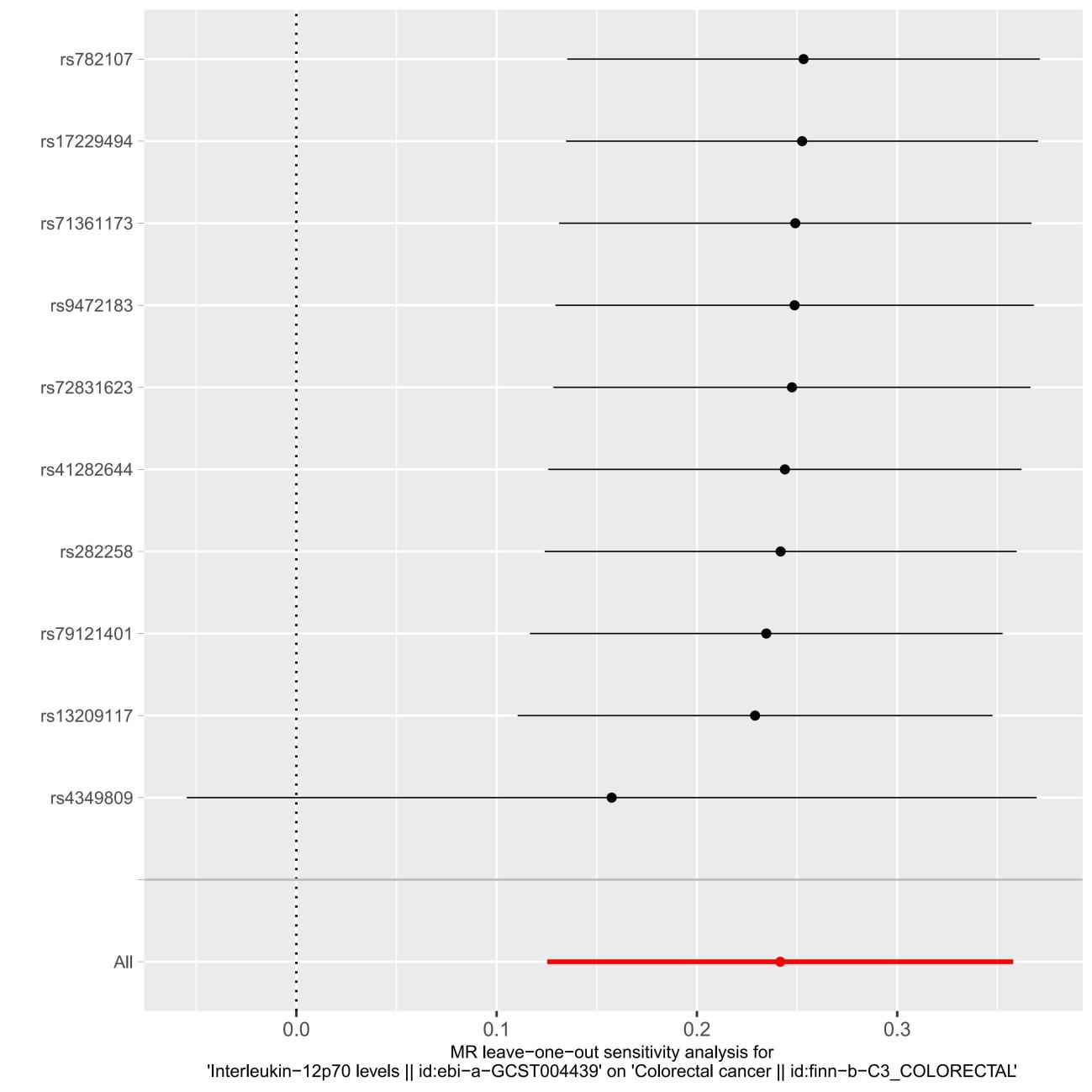


C. Leave-one-out plot of Interleukin-12p70 levels


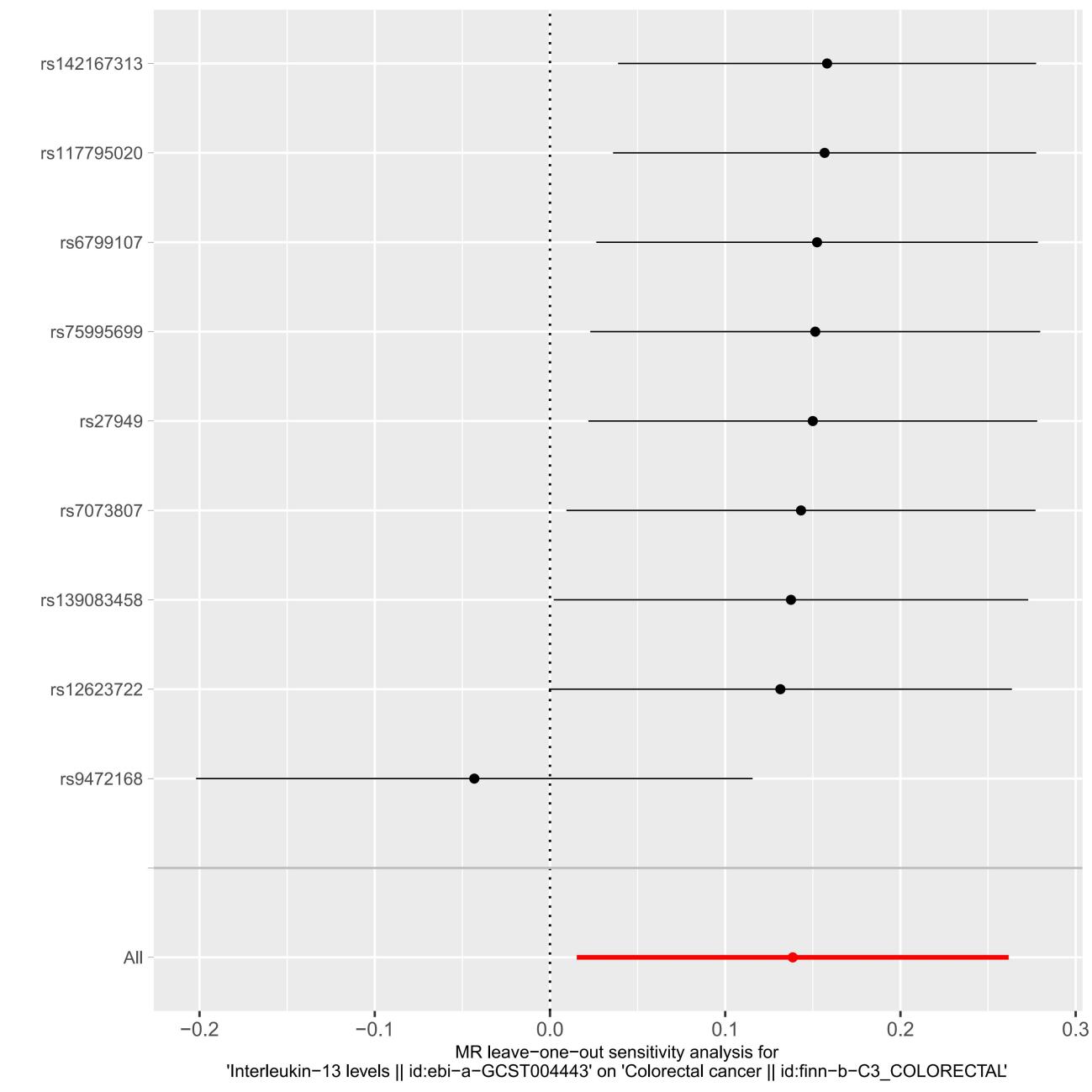


D. Leave-one-out plot of Interleukin-13 levels


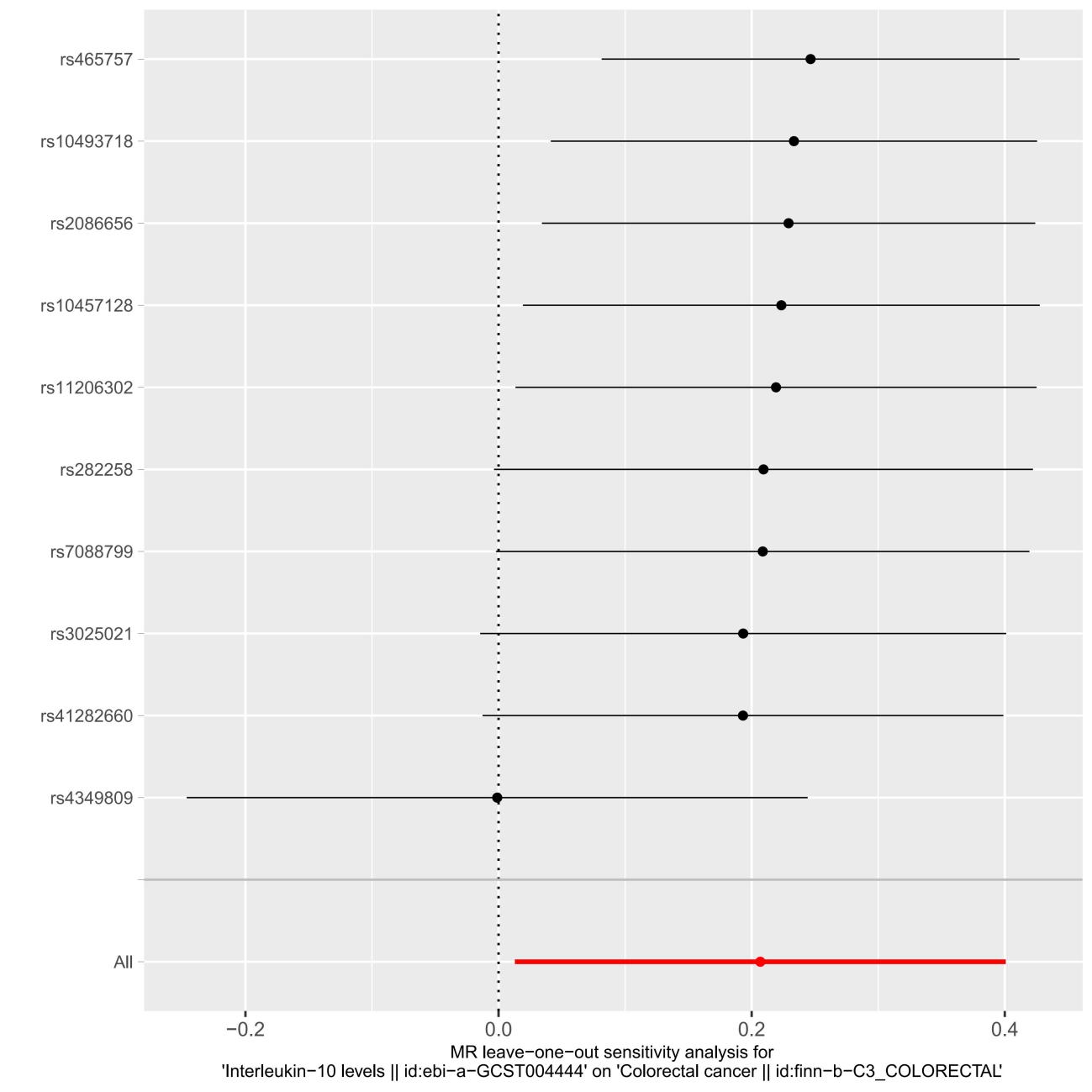


E. Leave-one-out plot of Interleukin-10 levels


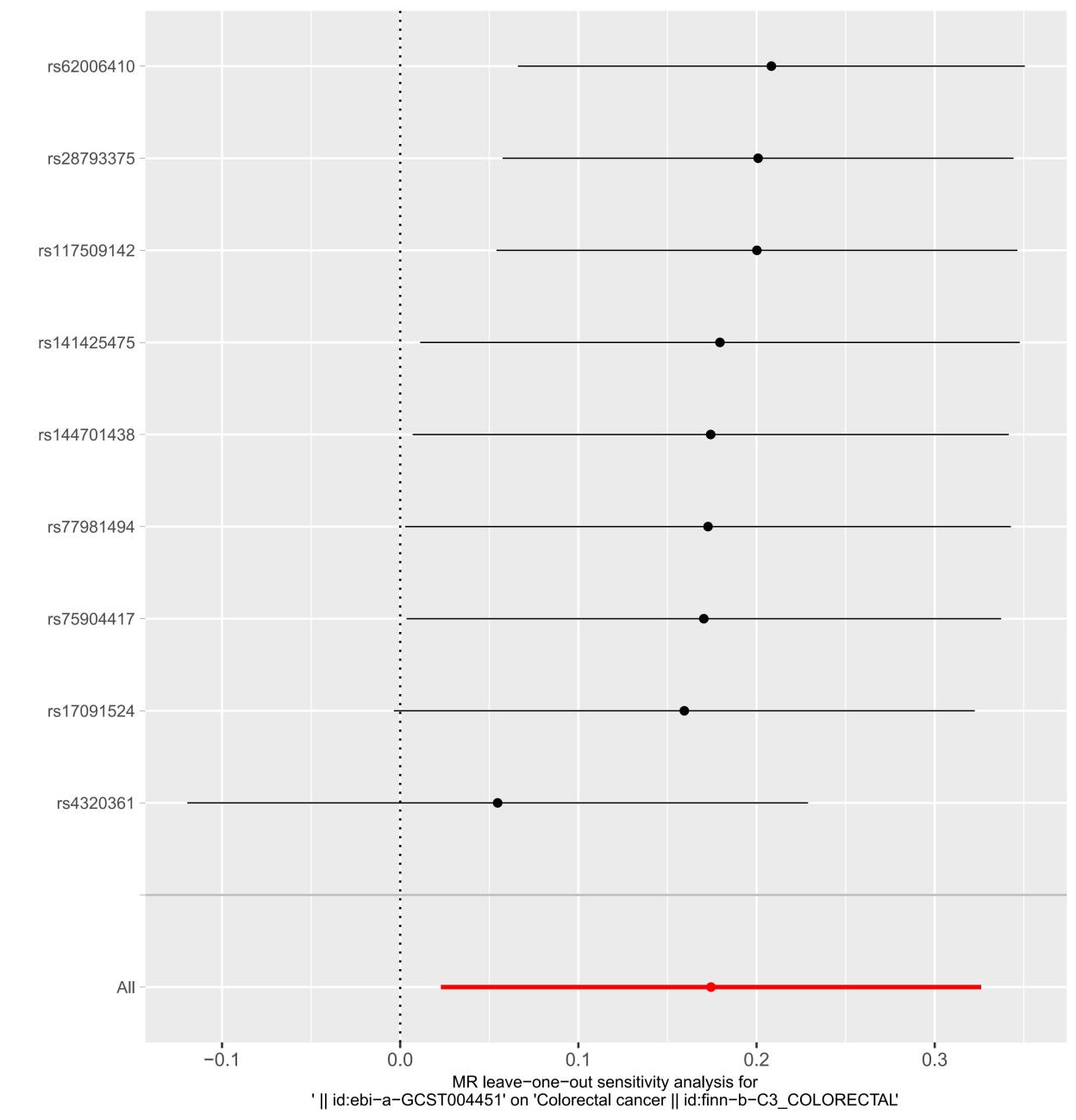


F. Leave-one-out plot of Interleukin-7 levels
